# Supplementary material for: 2D-GaN/AlN Multiple Quantum Disks/Quantum Well Heterostructures for High-Power Electron-Beam Pumped UVC Emitters
Source: Nanomaterials (Basel). 2023 Mar 16;13(6):1077. doi: 10.3390/nano13061077 (PMC10059987; doi:10.3390/nano13061077)
Supplement: Supplementary file 1 [file nanomaterials-13-01077-s001.zip › nanomaterials-2266494-supplementary.pdf]

nanomaterials

MDPI

Supplementary

1. Estimation of excess Ga and Al accumulated during QW growth under different Me-rich conditions.

During the growth of MQW structures under Me-rich conditions with various Ga/N_2* flux ratio the following typical growth parameters were used. MQW-2.2-S structure was grown at the growth fluxes of Ga=1.00 ML/s, N_2*=0.464 ML/s and the duration of the growth of QW with a nominal thickness of 1.5ML t_{QW}=3.2s. Substrate temperature was T_S=691°C and in accordance with [1] the equilibrium Ga desorption flux at this temperature was estimated as Ga^{DE} = 0.22ML/s. These fluxes provided the strong Ga-rich growth conditions of GaN: (Ga - N_2*) > Ga^{DE} and the effective Ga-desorption flux Ga^D = Ga^{DE}.Thus, the excess Ga accumulated during the QW growth equals

$$E^{Ga} = (Ga - Ga^D - N_2^*)\cdot t_{QW} = 1.0\text{ML} \qquad \text{(S1)}$$

MQW-1.1-S structure was grown at the growth fluxes of Ga = 0.50 ML/s, N_2* = 0.450ML/s and the duration of the growth of QW with a nominal thickness of 1.5ML t_{QW}=3.1s. At the same substrate temperature (691°C), the fluxes used ensured the weak Ga-rich conditions (Ga – N_2*) < Ga^{DE} , at which the Ga desorption flux was equal to Ga^D = (Ga – N_2*) = 0.5 – 0.45 = 0.05 ML/s and excess Ga did not accumulate.

2. Calculation of the stress in the 400×{$GaN_{1.5}$/AlN_{16}} MQW structure coherently grown on AlN layer

An average Al-content (x) in the 400×{$GaN_{1.5}$/AlN_{16}} MQW structure was calculated by the equation x=$d_{BL}/(d_{QW} + d_{BL})$, which at the used thicknesses d_{GaN}=1.5ML and d_{AlN}=16 gave the value x=0.91. Vegard's law was used for the calculations of the a-lattice constant of the equivalent ternary alloy as $a_{AlGaN}=a_{AlN}\cdot x + a_{GaN}\cdot(1\text{-}x)$=3.118 Å, where a-lattice constants for binary alloys were a_{AlN} = 3.111 Å, a_{GaN} = 3.189 Å [2]. Using these data, the biaxial strain in the coherently grown $Al_xGa_{1-x}N$/AlN heterostructure was estimated as $\varepsilon_{xx} = (a_{AlN} - a_{AlGaN})/a_{AlGaN}$ = -0.00221. In the same way, the biaxial elastic modulus of $Al_xGa_{1-x}N$ (was calculated as $M_{AlGaN} = M_{AlN}x + M_{GaN}(1\text{-}x)$ = 472 GPa with the following reference data for biaxial elastic modulus AlN and GaN: and M_{AlN} = 471 GPa [3], M_{GaN} = 479 GPa [4], which was used to estimate the biaxial stress in this coherently grown heterostructure as $\sigma_{xx} = M_{AlGaN}\times\varepsilon_{xx}$ = 1.0 GPa.

3. The SEM image of this structure with clearly visible cracks

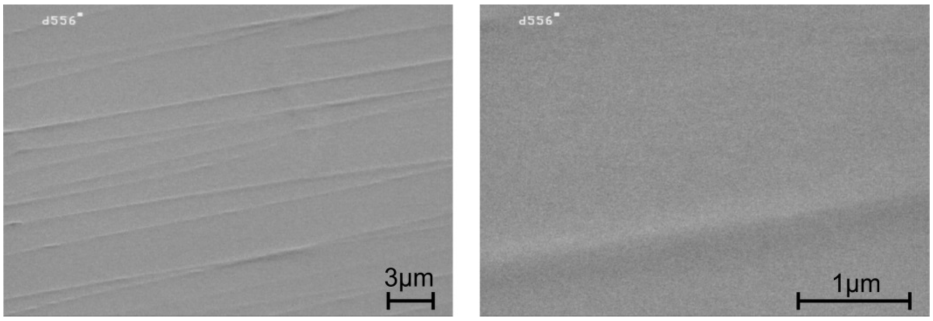

Figure S1. SEM images of structure MQW-2.2-D_1 obtained at various magnifications.

4. The (105) RSMs of {$GaN_{1.5}/AlN_{16}$} (N=50,100) MQW structures grown at various Ga/N_2* flux ratio

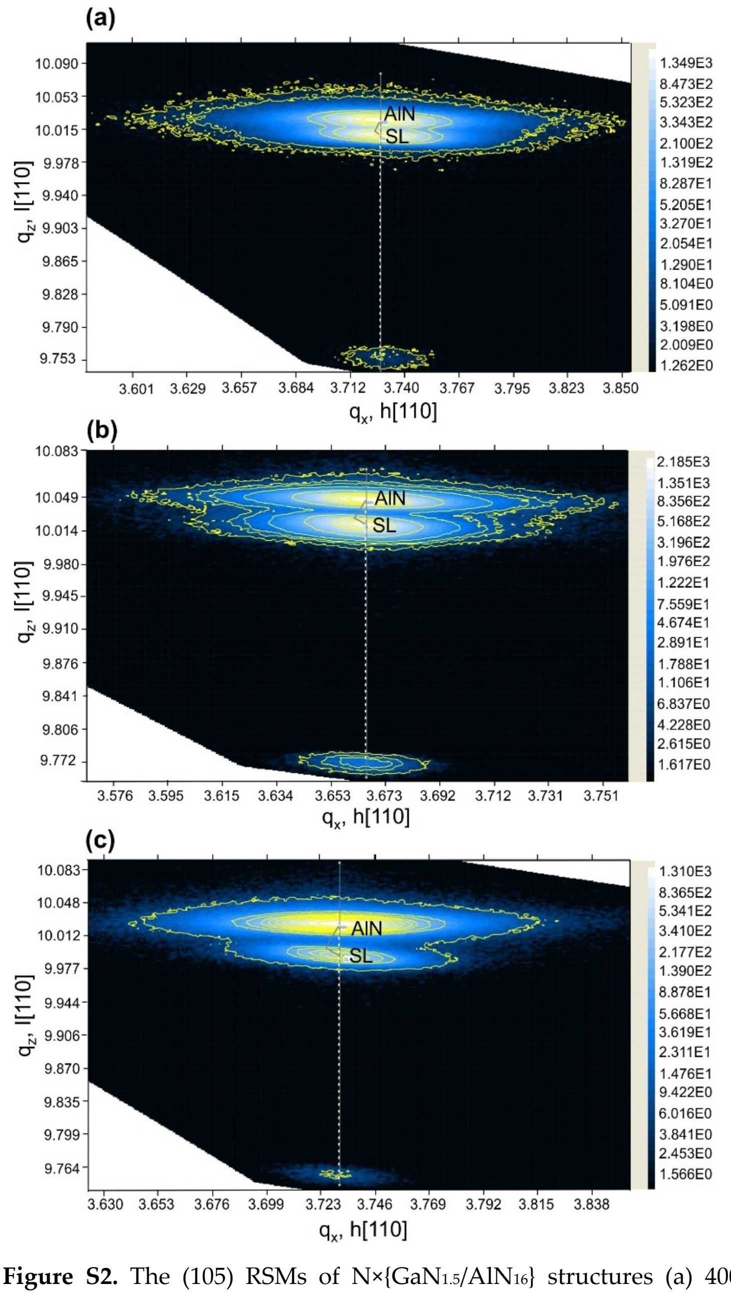

Figure S2. The (105) RSMs of N×{$GaN_{1.5}/AlN_{16}$} structures (a) 400×QW-1.5-1.1-S grown under Ga/N_2* =1.1; (b) 400×QW-1.5-2.2-S (c) 400×QW-1.5-2.2-S (both (b) and (c) were grown under Ga/N_2* =2.2).

1. Jmerik, V.N.; Nechaev, D.V.; Ivanov, S.V. Kinetics of metal-rich PA molecular beam epitaxy of AlGaN heterostructures for mid-UV photonics. In *Molecular Beam Epitaxy: From Research to Mass Production*, 2nd ed.; Henini, M., Ed.; Elsevier Inc.: Amsterdam, The Netherlands; Oxford, UK; Cambridge, MA, USA, 2018; pp. 135–179. https://doi.org/10.1016/B978-0-12-812136-8.00008-6.
2. Ferreyra, R.A.; Zhu, C.; Teke, A.; Morkoç, H. Group III Nitrides. In *Springer Handbook of Electronic and Photonic Materials*; Kasap, S., Capper, P., Eds.; Springer International Publishing: Cham, Switzerland, 2017. https://doi.org/10.1007/978-3-319-48933-9_31.
3. Wright, A. Elastic properties of zinc-blende and wurtzite AlN, GaN, and InN. *J. Appl. Phys*. **1997**, *82*, 2833–2839. https://doi.org/10.1063/1.366114.
4. Polian, A.; Grimsditch, M.; Grzegory, I. Elastic constants of gallium nitride. *J. Appl. Phys*. **1996**, *79*, 3343–3344. https://doi.org/10.1063/1.361236.
